# Open-Ended Coaxial Probe Measurements of Complex Dielectric Permittivity in Diesel-Contaminated Soil during Bioremediation

**DOI:** 10.3390/s20226677

**Published:** 2020-11-22

**Authors:** Andrea Vergnano, Alberto Godio, Carla Maria Raffa, Fulvia Chiampo, Jorge A. Tobon Vasquez, Francesca Vipiana

**Affiliations:** 1 Department of Environment, Land and Infrastructure Engineering, Politecnico di Torino, Corso Duca degli Abruzzi 24, 10129 Torino, Italy; alberto.godio@polito.it; 2 Department of Applied Science and Technology, Politecnico di Torino, Corso Duca degli Abruzzi 24, 10129 Torino, Italy; carla.raffa@polito.it (C.M.R.); fulvia.chiampo@polito.it (F.C.); 3 Department of Electronics and Telecommunications, Politecnico di Torino, Corso Duca degli Abruzzi 24, 10129 Torino, Italy; jorge.tobonvasquez@polito.it (J.A.T.V.); francesca.vipiana@polito.it (F.V.)

**Keywords:** open-ended coaxial probe, complex dielectric permittivity, diesel oil, bioremediation, contaminated soil

## Abstract

In the bioremediation field, geophysical techniques are commonly applied, at lab scale and field scale, to perform the characterization and the monitoring of contaminated soils. We propose a method for detecting the dielectric properties of contaminated soil during a process of bioremediation. An open-ended coaxial probe measured the complex dielectric permittivity (between 0.2 and 20 GHz) on a series of six soil microcosms contaminated by diesel oil (13.5% V_oil_/V_tot_). The microcosms had different moisture content (13%, 19%, and 24% V_w_/V_tot_) and different salinity due to the addition of nutrients (22 and 15 g/L). The real and the imaginary component of the complex dielectric permittivity were evaluated at the initial stage of contamination and after 130 days. In almost all microcosms, the real component showed a significant decrease (up to 2 units) at all frequencies. The results revealed that the changes in the real part of the dielectric permittivity are related to the amount of degradation and loss in moisture content. The imaginary component, mainly linked to the electrical conductivity of the soil, shows a significant drop to almost 0 at low frequencies. This could be explained by a salt depletion during bioremediation. Despite a moderate accuracy reduction compared to measurements performed on liquid media, this technology can be successfully applied to granular materials such as soil. The open-ended coaxial probe is a promising instrument to check the dielectric properties of soil to characterize or monitor a bioremediation process.

## 1. Introduction

Hydrocarbon biodegradation can cause changes in soil geophysical properties, like dielectric permittivity or electrical conductivity, for many reasons. The first and most evident is that, when hydrocarbon contamination is reduced, water or air takes its place. In this way, the relative quantities of the substances composing the soil matrix change, affecting the geophysical properties of the bulk [1]. More complex phenomena occur when biological processes are going on in soils. Soil weathering can induce variations in electrical conductivity [2], and the biological production of extracellular polymeric substances, known as biofilms, can cause changes in soil porosity as well as grain surface electrical interactions [3].

Monitoring geoelectric properties in a contaminated site can be a useful tool to understand the biodegradation processes in progress [4,5]. In bioremediation contexts, geophysical techniques have been commonly applied, such as electrical resistivity tomography, self-potential measurements, and acoustic impedance measurements [3,6,7] at the field scale. The application of geophysical methodologies for on-site monitoring of organic contaminants is widespread [8,9,10]. The monitoring over time (time-lapse) of changes in the chemical-physical characteristics of the soils is based on the application of geophysical sensors sensitive to changes in electrical and electromagnetic parameters. The most sensitive methodologies are based on the analysis of the complex electrical conductivity observed in the frequency domain and, more generally, on the analysis of induced polarization phenomena. Koroma et al. [11] verified the sensitivity of induced polarization measures in the frequency domain to the variation over time of the degradation-related effects in soil contaminated by hydrocarbons. This topic has been addressed at the laboratory scale and in the field [12,13].

The development of geophysical sensors for the quantitative evaluation of the effective degradation of organic compounds in the soil remains particularly challenging, especially for the complexity of the biological and chemical phenomena that are responsible for the variations of the geophysical parameters. The most significant results are obtained through the integration of electrical and electromagnetic methodologies, jointly with the biological and geochemical tests of soil and groundwater [14]. New insights about the relationships between the biogeochemical parameters and the geophysical signature could be achieved through laboratory investigation [15,16]. At the laboratory scale, the investigation of dielectric properties of materials has been often performed using resonant cavities and transmission lines [17,18,19]. Resonant cavities are designed for single-frequency measurements of dielectric values, but multi-frequency measurements may outline the dielectric properties of soil more comprehensively. Transmission line techniques need large samples, which makes the handling of granular materials difficult. Both techniques are widely used to measure low-lossy materials, but contaminated soil can be lossy because of the presence of water. Therefore, the characteristics of contaminated soil reveal the need for more suitable and convenient alternatives.

The use of open-ended coaxial probes (OECP) has been very common in biological and biomedical applications [20,21] to test agricultural products [22] and, in general, to measure lossy materials [23]. Its application has been uncommon in soil characterization and monitoring. It was tested in contaminated soils by Godio [24], who also reviewed the theoretical background of electrical properties of soil with a focus on the dielectric response of retention water (bound water). Another research focused on the OECP measurement of different soil samples with different water content and salinity, pointing out that the imaginary component of dielectric permittivity, at low frequencies, is particularly sensitive to variations in the water salinity [25]. Models like the Complex Refractive Index Model (CRIM) have been developed and widely applied [26] to estimate the soil volumetric water content or diesel oil content from measurements of dielectric permittivity.

Previous laboratory-scale research investigated the use of Time Domain Reflectometry (TDR) probes in contaminated soils [1,27]. A comparison between TDR and open-ended probes was carried out by Skierucha et al. [28], finding that, at frequencies around 1 GHz, the two technologies give similar dielectric permittivity results. The open-ended coaxial probe provides more detailed information about the dielectric properties than TDR sensors. Moreover, having the possibility to measure a spectrum of frequencies, the OECP may detect eventual different polarization phenomena, which are responsible for variations in both the real and imaginary component of the dielectric permittivity.

In such a context, we applied the open-ended coaxial probe technology to contaminated soil during a process of bioremediation. Our goal was to test their use as a support instrument for geophysical surveys on contaminated sites. First, we calibrated the sensor in known liquid media. Then, we set-up a series of measurements in six soil microcosms, contaminated with diesel oil, and realized with favorable bioremediation conditions for aerobic autochthonous microorganisms. The microcosms had different moisture content and salinity, so we were able to check the accuracy issues arising by using this sensor in a granular material such as soil. We discussed the results on the basis of previous research in which we have performed physico-chemical analyses (e.g., gas chromatography and fluorescein diacetate production) regarding the diesel degradation and the biological processes on the same soil [29].

In the following sections, we first outline the theory of the dielectric properties of materials with a focus on the models that describe the soil matrix. Then, we present the open-ended coaxial probe and our design of the soil microcosms. We show the results, discussing the reliability of the methodology to assess a bioremediation process. Finally, based on our research, we compare the open-ended coaxial probe with another technique used in this field, known as the Time Domain Reflectometry).

## 2. Theoretical Background

Dielectric properties of a medium can be described by the complex dielectric permittivity (*ε*), which is a quantity composed of a real and an imaginary component. The real part represents the electric field that the material generates when an external electric field (***E***) is applied, called polarization, while the imaginary component describes the dielectric and the conductive losses [27]. This charge displacement can be described by the electric flux density of the electrical displacement vector (***D***), and it is related to dielectric permittivity.

(1) D=εE

The real and the imaginary component compose the complex dielectric permittivity (*ε_r_*).

(2) $$\varepsilon_{r}=\varepsilon_{r}^{\prime }+i(\varepsilon_{r}^{\prime \prime }+\frac{\sigma_{dc}}{2\pi f\varepsilon_{v}})$$

where the dimensionless relative permittivity (or dielectric constant) is defined as:

(3) $$\varepsilon_{r}=\frac{\varepsilon }{\varepsilon_{vacuum}}$$

The first term of the imaginary component (*ε_r_″*) represents the dielectric losses, related to the vibration or rotation of the molecules. The second term represents the losses due to conductivity [30].

The frequency dependence of complex dielectric permittivity is due to the non-instantaneous polarization response of a material to the applied electric field, so that ***D*** is not linearly related to ***E*** as in Equation (1), but ε=ε(f), where *f* is the frequency. Each material exhibits a characteristic relaxation time (*τ*), that is related to the time required for dipoles to orientate, according to the external electric field. The correspondent relaxation frequency is:

(4) $$f_{c}=\frac{1}{2\pi \tau }$$

**Figure 1:**
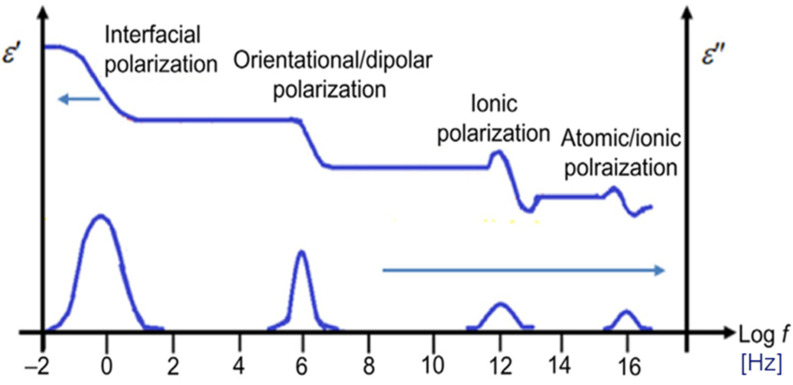
Different polarization effects in solid materials [32].

Some materials can exhibit more than one relaxation time, as they are subjected to more than one polarization mechanism (Figure 1). Orientation or dipolar polarization, atomic polarization, and electronic polarization are the most common [31].

Considering the simplest case of single relaxation time (*τ*), the frequency dependence of permittivity can be described with the Debye equation.

(5) $$\varepsilon \left(\omega \right)=\frac{\varepsilon_{dc}-\varepsilon_{\infty }}{1+i\omega \tau }$$

that can be divided into:

(6) $$\varepsilon^{\prime }=\varepsilon_{\infty }+\frac{\varepsilon_{dc}-\varepsilon_{\infty }}{1+\omega^{2}\tau^{2}}$$

(7) $$\varepsilon^{\prime \prime }=\frac{\left(\varepsilon_{dc}-\varepsilon_{\infty }\right)\omega \tau }{1+\omega^{2}\tau^{2}}$$

where *ε_dc_* is the direct current or zero frequency permittivity, *ε_∞_* is the infinite frequency permittivity, ω=2πf, and *τ* is the relaxation time.

At low frequencies, the polarization mechanism is fast enough to keep pace with the oscillations of the electric field, so that the real component is fairly constant. It can be observed that the dielectric losses (*ε_r_″*) increase with direct proportionality to the frequency. At frequencies near relaxation, *ε_r_″* continues to increase but the storage capacity (*ε_r_’*) decreases because the polarization cannot fully develop, and a phase lag between the electric field and the dipole alignment occurs. At higher frequencies, both real component and dielectric loss component drop off, as the oscillations of the electric field happen at too great speed to influence the orientation polarization mechanism. At the highest frequencies, other mechanisms such as atomic and electronic polarization will take place.

The Debye equations were semi-empirically modified by some authors, who studied the dielectric properties of polymers, to better fit real observations, reviewed by Psarras [32].

The dielectric permittivity, in a multi-phase material as soil, depends on the dielectric properties of the various phases. The porosity of soil can be filled by water, air, and other compounds in fluid phases (e.g., contaminants), more often by a combination of them. The CRIM model [26] was developed to provide a simple correlation between the measured real dielectric permittivity and the percentage of the various phases in a soil.

(8) $$\varepsilon_{b}^{\alpha }=\varepsilon_{s}^{\alpha }\left(1-\phi \right)+[\varepsilon_{w}^{\alpha }S_{w}+\varepsilon_{o}^{\alpha }S_{o}+\varepsilon_{a}^{\alpha }\left(1-S_{w}-S_{o}\right)]\phi$$

where ϕ is the porosity, *S* is the saturation, and subscripts *b*, *s*, *w*, *a,* and *o* refer to bulk, soil grains, water, air, and oil, respectively. The exponent α has values around 0.5.

Archie’s Law, instead, allows for the calculation of the electrical conductivity of a soil matrix from the values of water-solution conductivity and porosity.

(9) $$\sigma_{t}=\frac{1}{a}\sigma_{w}\varphi^{n}S_{w}^{m}$$

where *n* is the cementation exponent, ranging from 1.3 in sands to 2 in consolidated rocks, *a* is the tortuosity factor or lithologic factor, and *m* is the saturation exponent, equal to 2 in non-polluted soils, up to 3–4 in the presence of hydrocarbons [33].

## 3. Materials and Methods

### 3.1. Set-Up of Microcosms

The study was carried out in six jars filled with 200 g of soil, water, and diesel oil, called microcosms. They were supposed to simulate contaminated soil in which biological processes of degradation can happen, as demonstrated by previous research [15].

**Figure 2:**
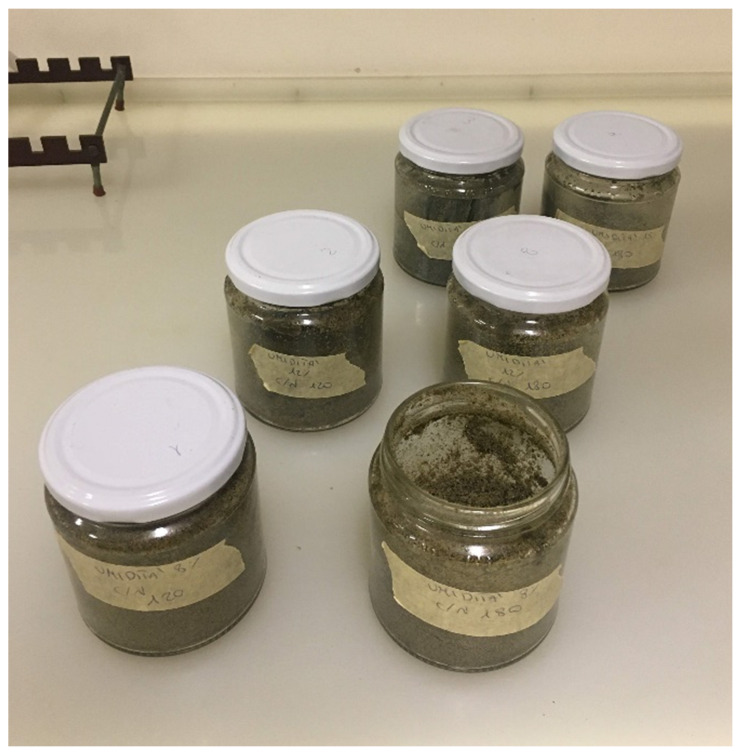
Contaminated soil microcosms.

The sandy soil was sieved to obtain a particle size distribution between 0.15 and 2 mm. It was contaminated with the addition of diesel oil and bio-stimulated by adding a nutrient solution in the form of Mineral Salt Medium for Bacteria (MSMB) [29]. Microcosms had a different volumetric water content (13% to 19%–24%) and amount of nutrients (Carbon/Nitrogen ratio, C/N = 120, 180), in any combination of them, as described in Table 1 and shown in Figure 2. The jars were kept sealed during the whole process to avoid evaporation, except when aeration was provided by manual mixing two to three times per week.

### 3.2. Open-Ended Coaxial Probe

The open-ended coaxial probe technology allows us to measure separately the real and the imaginary component of the complex dielectric permittivity, analyzing the reflection coefficient of an electromagnetic signal at the probe-sample interface and applying inversion algorithms. A coaxial probe consists of a truncated coaxial cable whose surface will be in contact with the measured dielectric sample. The electromagnetic field that travels on the coaxial transmission line will encounter a mismatch in the probe-sample interface and the wave will be partially reflected. As explained in a study by Komarov et al. [34], “the incident principal transverse electric and magnetic (TEM) field mode, generated by the network analyzer, propagates along the waveguide. In the vicinity of the aperture, the TEM wave is getting distorted, and the vector of the electric field starts gaining a component perpendicular to the aperture plane. The energy of the incident wave is partially radiated into the dielectric half-space and partially reflected back to the coaxial waveguide. The complex magnitude of the reflected wave significantly depends on the dielectric properties of the tested material” [34]. The reflection coefficient (measured by a Vector Network Analyzer) of the probe-sample system depends on the dielectric properties of the sample. Therefore, the measurement of the reflection coefficient is a measurement of the dielectric properties of the material. The extraction of the latter data can be obtained from the actually measured reflection coefficients. In general, the measurement is performed in a frequency sweep instead of a single frequency approach. Summarizing, the flanged open-ended coaxial probe can be used for measurements of the material’s complex dielectric permittivity. Inversion algorithms and full-wave solutions have been widely studied [35,36,37,38,39], and a good explanation of the different mathematical and computational processes is provided by Reference [34].

The advantages of this probe are related to its simplicity including the probe having to touch the interface during the measurement time with no need for sample destruction for most of the dielectric materials. In addition, it performs the acquisition over the complete frequency band data with no alteration of the probe nor of the sample. The measurement time for the complete band depends on the network analyzer sweep time, and it is in the range of a couple of seconds. The manufacturer of the probe is Keysight, and the model is 85070D Dielectric Probe Kit, composed of an open-ended coaxial probe (to be connected to a network analyzer) and its software.

In this particular case, the diameter of the probe is 3.2 mm (19 mm considering the large flange around the truncated coaxial) and requires a sample of at least 20 mm. The probe has a flange around the external conductor to avoid field leakage and to contain most of the field lines that cross the sample. A thickness requirement is determined by $\frac{20}{\sqrt{\epsilon_{r}}}mm$ (as in the datasheet [34]), considering the expected dielectric values. In the microcosms under analysis, these requirements were satisfied widely.

The large flange of the probe makes it easier to measure our semi-solid, granular material. Due to the heterogeneity of the size of soil grains, the repeatability of the measurements could vary, especially considering that the limit region for granularity recommended by the probe datasheet is 0.3 mm. Multiple measurements in different points could confirm homogeneity on the density and independent behavior with respect to the granularity in our case, and we propose the approach to confirm repeatability. The probe suits non-magnetic, isotropic, and homogeneous materials. Homogeneity can be an issue of the same nature as granularity. It is important to highlight that the typical accuracy declared by the manufacturer is 5%, but it depends on the properties of the material under tests. At lower frequency, for low $\epsilon_{r}$ values, this accuracy can reach values above 10% [40].

The calibration of the probe requires the measurement of samples where the electromagnetic properties are well known, as distilled water, air (leaving the probe open), and a short circuit standard. This three-term calibration corrects the directivity, tracking, and source match errors of the reflection measurement, removing the systematic errors. Special care must be taken in the pressure and stillness of the probe during the measurement time, as probe stability and air gaps can introduce an error for the measurement.

The open-ended coaxial probe was selected because it is capable of measuring complex dielectric permittivity in a broad frequency range. In particular, the ability to distinguish the real and imaginary components makes it interesting to be applied in contaminated soil, where different biological processes can influence both.

The open-ended coaxial probe was connected to a network analyzer and the head of the sensor was put in direct contact with the soil, measuring complex dielectric permittivity in a 0.2–20 GHz frequency range. The measurements were performed at the beginning of the set-up and, after 130 days, to control if the evolution of biodegradation happening in microcosms induced variations in geoelectric properties. Each sample was stirred to make them homogeneous, and the measure was repeated 10 times, moving the probe in different areas of the sample surface. Temperature calibration was performed.

**Figure 3:**
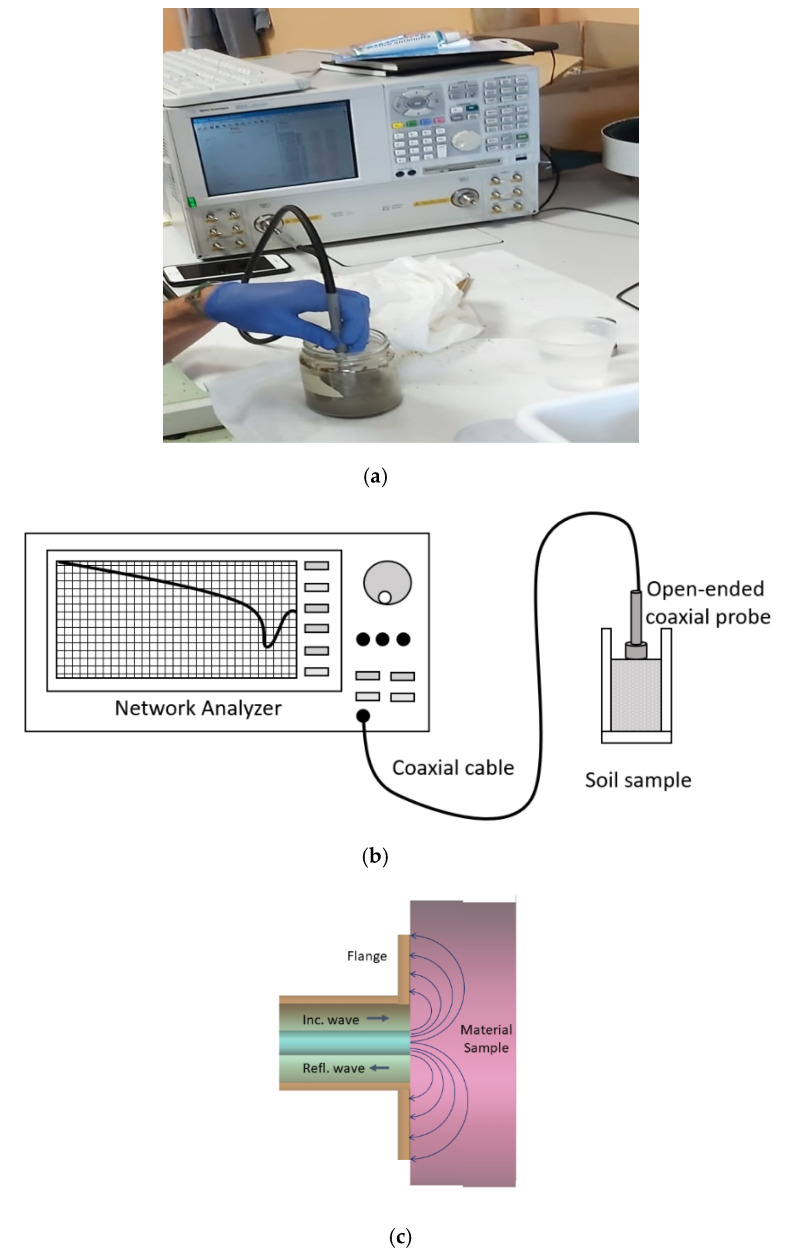
Network analyzer and dielectric probe measuring the permittivity of contaminated soil: (**a**) instrument, (**b**) apparatus scheme, and (**c**) open-ended coaxial probe scheme.

Figure 3 shows how the end of the probe was put in contact with the soil.

## 4. Results

**Figure 4:**
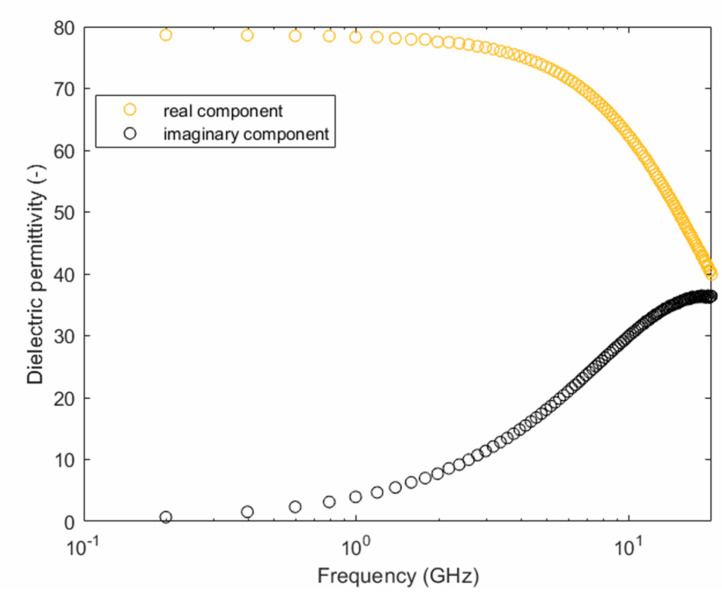
Real and imaginary dielectric permittivity measured on distilled water.

The complex permittivity spectrum of distilled water was measured with the open-ended coaxial probe during the calibration process. Figure 4 shows in orange the real component, and, in black, shows the imaginary one.

**Figure 5:**
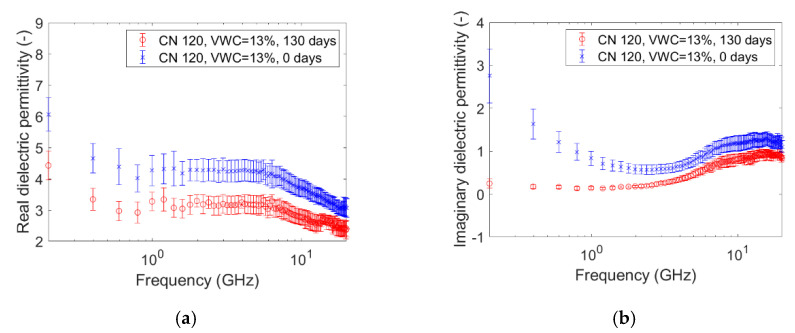
Complex dielectric permittivity before bioremediation and after 130 days: real part (**a**), imaginary part (**b**). Microcosm: C/N = 120, volumetric water content = 13%.

**Figure 6:**
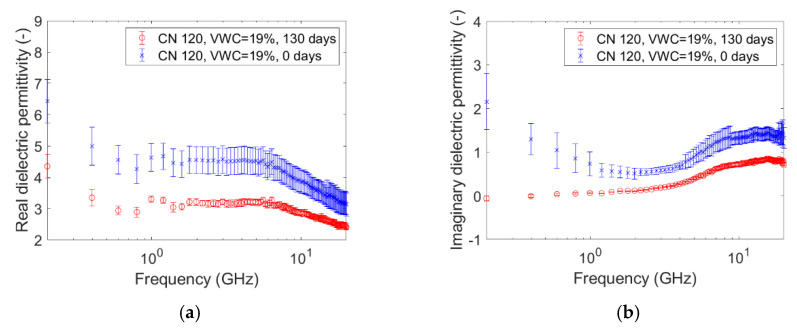
Complex dielectric permittivity before bioremediation and after 130 days: real part (**a**), imaginary part (**b**). Microcosm: C/N = 120. Volumetric water content = 19%.

**Figure 7:**
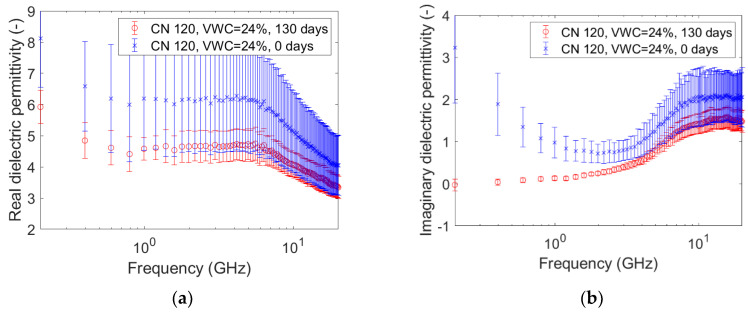
Complex dielectric permittivity before bioremediation and after 130 days: real part (**a**), imaginary part (**b**). Microcosm: C/N = 120. Volumetric water content = 24%.

**Figure 8:**
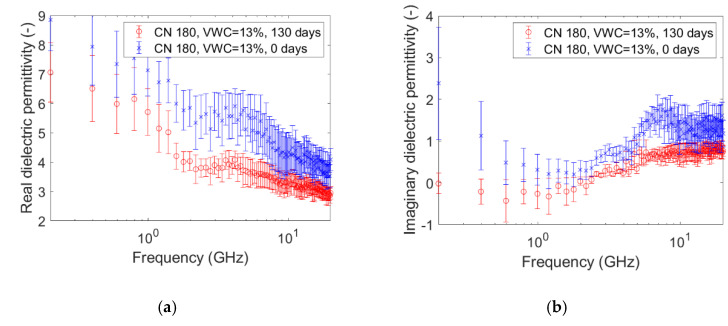
Complex dielectric permittivity before bioremediation and after 130 days: real part (**a**), imaginary part (**b**). Microcosm: C/N = 180. Volumetric water content = 13%.

**Figure 9:**
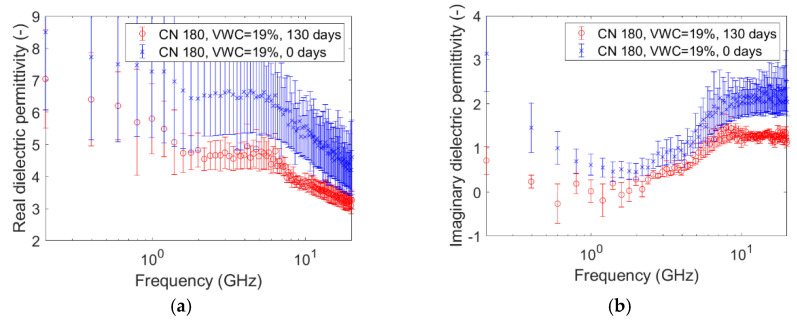
Complex dielectric permittivity before bioremediation and after 130 days: real part (**a**), imaginary part (**b**). Microcosm: C/N = 180. Volumetric water content = 19%.

**Figure 10:**
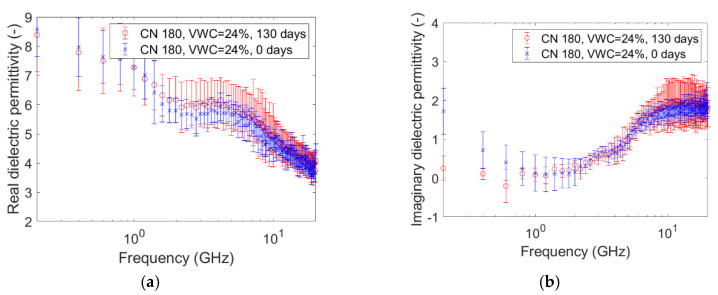
Complex dielectric permittivity before bioremediation and after 130 days: real part (**a**), imaginary part (**b**). Microcosm: C/N = 180. Volumetric water content = 24%.

The graphs pictured in Figure 5, Figure 6, Figure 7, Figure 8, Figure 9 and Figure 10 are related to the measurements performed on the soil microcosms at time 0 and after 130 days. The microcosms had different water contents and two different values of the Carbon/Nitrogen (C/N) ratio. The pictures show the real part (*ɛ*’) and the imaginary part (*ɛ*″) of the dielectric permittivity versus the frequency. The permittivity values at the initial stage of contamination (in blue) are compared with those after 130 days (in red). The main observations are summarized in Table 2.

The spectra observed after 130 days (in red) always show greater values than the reference ones (at time zero, in blue) for both the components. This is not valid for the only case reported in Figure 10a,b, where the spectra at the two times overlap rather well. Furthermore, it can be observed that the imaginary component substantially decreased at low frequencies in all microcosms.

**Figure 11:**
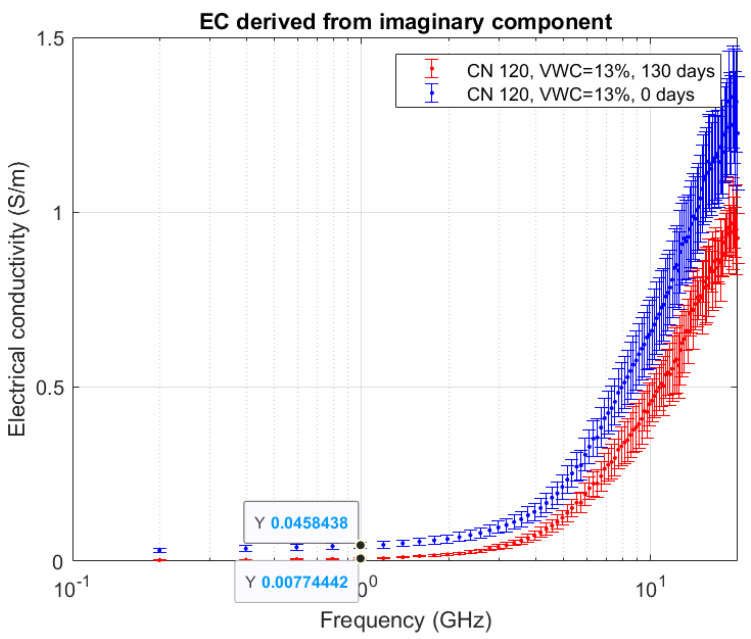
Electrical conductivity derived from the mean of the imaginary component of dielectric permittivity. Microcosm: C/N = 120. Volumetric water content = 13%.

The imaginary component is linked to the electrical conductivity, according to Equation (2). Figure 11 shows the electrical conductivity of one of the microcosms as a representative example of all. The values highlighted are at 1 GHz.

The dataset containing the raw data elaborated in this section is available in the Supplementary Materials.

## 5. Discussion

### 5.1. Preliminary Calibration and Accuracy

The measurement on the distilled water sample showed values of permittivity similar to those found in the literature [31,41,42]. The complex permittivity of water in the frequency range from 0.2 GHz to 20 GHz is influenced by the orientation polarization phenomenon. The real part starts to decrease at a frequency greater than 1-2 GHz because the dipole orientation of its molecules is not as fast as the directional change of the electric field. This causes dielectric losses that are indicated by the corresponding increase of the imaginary part. However, the frequency range of our measurement was not wide enough to signal the whole phenomenon, nor has it demonstrated other phenomena like atomic or electronic polarization, which happen at higher frequencies. The measurement on water, alongside the measurement on air and short circuit (provided by the manufacturer), was analyzed by the commercial software to calibrate the instrument.

Compared to the calibration measurements on water samples, we observed a drop in accuracy in the measurements performed on contaminated soil (Figure 5, Figure 6, Figure 7, Figure 8, Figure 9 and Figure 10). The permittivity values of replicas were often fairly different from each other, and this led to a high standard deviation in some cases (Figure 7a, Figure 8a and Figure 9a). This showed that granular materials like sandy soil can cause inaccuracy problems due to the small area of sensitivity of the open-ended probe. The grain size of the soil used in this study was 0.15–2 mm. The gaps between the grains were sufficiently wide to moderately affect the repeatability of the measurement. The standard deviation, however, decreased from the initial conditions to 130th-day measurements. This is not easily explained, but we speculated that the soil changed in some physical characteristics such as porosity. For instance, porosity changes could be related to the progressive removal of diesel and the formation of secondary products like biofilms. This phenomenon was previously observed by Davis et al. [3] who demonstrated that the electrical conductivity is sensitive to the physical-chemical properties at the grain-fluid interface, and that the imaginary conductivity component could be a good indicator of the microbial activity and biofilm formation in porous media. Another hypothesis is that the granular size distribution changed due to soil weathering, induced by the production of acid co-metabolites by biological activity, as observed by Atekwana [43].

### 5.2. Analysis of Experimental Results

The real part of the dielectric permittivity was shown to be frequency-dependent (Figure 5a, Figure 6a, Figure 7a, Figure 8a, Figure 9a and Figure 10a) with an inverse proportionality similar to that of water. However, its trend was more complex. It decreased from 0.2 GHz to about 1 GHz, then it remained constant until about 6 GHz, and then it decreased again. This behavior could be explained by the evidence of an interfacial polarization effect, typical of solid materials, and illustrated in Figure 1 [32]. We observed that, in the frequency range in which it remained constant, the real component values ranged from 3 to 7. This is acceptable to follow in the broad terms of the CRIM model (Equation (8)), but not sufficiently accurate to distinguish correctly two microcosms with different amounts of water (for example, see Figure 5a, Figure 6a and Figure 7a). Therefore, all the following calculations on the values of real or imaginary permittivity provided results in the correct order of magnitude, but cannot be considered as quantitatively accurate.

Nevertheless, comparing the real permittivity of microcosms before and after 130 days of remediation, almost all microcosms showed a significant decrease (up to 2 units) at all frequencies. We explained it by assuming a decrease of the liquid components of the sample. The measurements of physical, chemical, and biological properties of the same microcosms have been performed during the 130 days of remediation, and have been extensively discussed by Raffa et al. [29], who demonstrated the effectiveness of the treatment in reducing the diesel concentration. In particular, the study reported an average diesel removal of about 65% (from 35% to 12% of diesel-oil saturation) after 130 days. However, in this range of values of water content, diesel content, and porosity, according to the CRIM model (Equation (8)), the observed decrease of the real permittivity cannot be caused only by the diesel removal. We speculated that a fraction of water evaporated during the mixing operations to provide oxygen, although the lack of accuracy of the instrument does not allow a meaningful quantitative calculation of the water loss, if any. In other words, the results of the CRIM model can be considered only as speculation about eventual water loss. The microcosm C/N = 180, VWC = 24% was an exception, showing no decrease over time, even if the accuracy issue must be considered again.

The trend of the imaginary part of the dielectric permittivity was more interesting. It showed a different behavior to the one measured on water. It initially decreased, and then increased after 2–3 GHz. At t = 0, the high values at low frequencies (around 200 MHz) were initially supposed to be caused by the interface polarization mechanism, which is in agreement with the suppositions made about the real component (see Figure 1). However, after 130 days, the 200-MHz imaginary permittivity tended to zero in all microcosms, so we rejected the first hypothesis and speculated a link between the imaginary component and the nutrient concentration. The nutrients were likely depleted by the biological activity, and this depletion caused a decrease of the dissolved salts in the pore volume water and, therefore, a decrease of the electrical conductivity, which is dependent on low-frequency imaginary dielectric permittivity (Figure 5b, Figure 6b, Figure 7b, Figure 8b, Figure 9b and Figure 10b). Another study already observed a strong dependence of imaginary permittivity at low frequencies on soil-moisture salinity, using the same technology [25]. This is also consistent with the findings pointed out in Reference [6], where the decrease of the imaginary conductivity was related to a decrease of the biomass activity or death of biomass because of nutrient limitations. The authors assumed that biomass reduction is caused by the accumulation of waste products (e.g., organic acids) and low pH conditions.

To assess the validity of the open-ended coaxial probe in bioremediation contexts, we checked possible correlations between these results and the results obtained in previous research about Time Domain Reflectometry (TDR) sensors [27]. We observed that the electrical conductivity measured directly with TDR sensors in similar polluted soil (but with less-salty nutrient solution) decreased from 0.006 to 0.004 S/m in about two months due to aerobic biological processes. For the current study, instead, the electrical conductivity was derived from the imaginary component of dielectric permittivity according to Equation (2), as shown in Figure 11. In particular, we focused on the values at 1 GHz because Skierucha et al. [28] compared TDR and OECP sensors in their research, and suggested that, at this frequency, the two instruments can be broadly compared. However, we point out that the two instruments use two very different technologies and their comparison is to be taken with care [44]. We found a value of 0.05 S/m at the beginning of the experiment, which is consistent with the electrical conductivity of the nutrient solution, according to Archie’s Law (a = 1, cementation index = 1.3, saturation exponent = 3). After 130 days, a strong decrease in electrical conductivity to about 0.007 was detected. In both OECP and TDR measurements, a marked decrease in the electrical conductivity was observed with the due differences in absolute values since the microcosms had different nutrient solutions.

### 5.3. Limitations

In the reclamation of a polluted site, and also in the preliminary lab-scale assessment, the choice of geophysical techniques and sensors is crucial for the needed outcomes. In previous research, we evaluated the use of Time Domain Reflectometry. In this study, we focused on the open-ended coaxial probe, which is a novelty in the bioremediation field. Each has its strengths and weaknesses. A TDR probe can measure the dielectric permittivity and the electrical resistivity simultaneously and continuously, making it suitable for a long-term monitoring task of water content and salinity. A single 12 V battery can provide enough power to supply multiple TDR probes for months, and their measurements can be stored in portable dataloggers. Its sensing volume is about 6 dm^3^ for a 12-cm TDR probe. As such, at the field scale, it provides only localized measurements, which can be used to calibrate the results of other techniques like Electrical Resistivity Tomography (ERT). At the lab scale, its sensing volume causes the need for big tanks or columns in which the fluid displacement can be great and may hamper the accurate sampling needed for further laboratory analyses. The open-ended coaxial probe, instead, is more focused on precise measurements at the lab scale, while it is not practical for field-scale monitoring. It provides measurements of complex dielectric permittivity over a wide frequency range, which are generally sufficient for a complete characterization of the soil’s electrical properties. The measurement is very localized (some cm^3^), and the accuracy suffers if the soil has a coarse granule size distribution. Our samples, in which the soil-grains diameter ranged from 0.15 to 2 mm, were slightly too coarse to ensure reliable accuracy. However, if loamy or silty soil is measured, or if larger-diameter probes are tested [45], this problem should be overcome. The higher cost of this technology makes it less suitable for multiple-samples monitoring at lab scale. However, it can be used successfully for preliminary analyses and for checking the results of other less-accurate and cheaper techniques.

## 6. Conclusions

The method allowed us to estimate both the electrical permittivity and conductivity in a wide frequency range to focus on various/certain phenomena of the degradation process. The real component of the complex dielectric permittivity was dependent on the moisture content and contaminant concentration, whereas the imaginary component was likely related to the decrease of the nutrients or some peculiar interaction between the solid phase and the liquid within the pore volume.

The adoption of the open-ended coaxial probe in the analysis of a bioremediation process in contaminated soil seems to be promising for monitoring the behavior of degradation processes. The challenging issue, namely the lack of accuracy when this probe is adopted in a porous and heterogeneous material, is acceptable if qualitative information on the trend of the removal process is the main goal. The quantitative interpretation of data (e.g., rate of removal of the contaminants) appears more challenging and is not yet resolvable because of the limitation of the method when porous solid materials are measured. Despite the accuracy issues, we were able to qualitatively check some significant differences in the observed geophysical properties, which are related to the soil matrix properties and the degradation process. Future directions will mainly involve the improvement in accuracy to develop a tool able to obtain quantitative information about the dielectric properties of soil. This instrument might be tested on slightly less coarse granular material, and larger-diameter open-ended coaxial probes might be tested and calibrated.

## Figures and Tables

**Table 1:** Main properties of microcosms.

Parameter (UM)	Value
Soil (kg)	0.2
Diameter of glass jars (cm)	7
Height of soil (cm)	~3
Total volume (l)	0.115
Porosity	0.4
Real density of soil (kg/m^3^)	2700
Apparent density of soil (kg/m^3^)	1620
Diesel oil (g)	13
Volumetric diesel oil content (l_oil_/l_total volume_)	13.5%
Particle size distribution (mm)	0.15–2
Volumetric water content (l_w_/l_total volume_)	13% to 19%–24%
Carbon/Nitrogen ratio (g/g)	120–180
Electrical conductivity of solution (S/m)	1.80 (C/N = 120)–1.21 (C/N = 180)

**Table 2:** Summary of complex dielectric permittivity measurements on the microcosms. Due to the accuracy issues, these values must be taken only as qualitative.

Microcosm:	C/N = 120, VWC = 13%	C/N = 120, VWC = 19%	C/N = 120, VWC = 24%	C/N = 180, VWC = 13%	C/N = 180, VWC = 19%	C/N = 180, VWC = 24%
Real part, t = 0, 1 GHz	4.3	4.7	6.2	7.8	7.3	7.2
Real part, t = 130, 1 GHz	3.3	3.4	4.7	5.7	5.8	7.2
Imag. Part, t = 0, 200 MHz	2.8	2.2	3.3	2.4	3.1	1.7
Imag. part, t = 130, 200 MHz	0.2	0	0	0	0.8	0.3

## Supplementary Materials

The following dataset are available online at https://www.mdpi.com/1424-8220/20/22/6677/s1.
